# 1,4a,7-Trimethyl-7-vinyl-1,2,3,4,4a,4b,5,6,7,9,10,10a-dodeca­hydro­phenanthrene-1-carboxylic acid

**DOI:** 10.1107/S1600536809013233

**Published:** 2009-04-25

**Authors:** Yu-xiang Chen, Zhen-dong Zhao, Yan Gu, Yu-min Wang

**Affiliations:** a Institute of Chemical Industry of Forest Products, Chinese Academy of Forestry, Nanjing, 210042, People’s Republic of China

## Abstract

The title compound, pimaric acid, C_20_H_30_O_2_, was isolated from a mixture of resin acids. There are three rings in the structure. The two cyclo­hexane rings have classical chair conformations with *trans*-fused ring junctions. The cyclo­hexene ring appears as a semi-chair.

## Related literature

For physical and spectral data relating to pimaric acid, see: Green *et al.* (1958 ▶); Harris & Sanderson (1948 ▶). For the biological activity of pimaric acid, see: Imaizumi *et al.* (2002 ▶); Rubio *et al.* (2005 ▶).
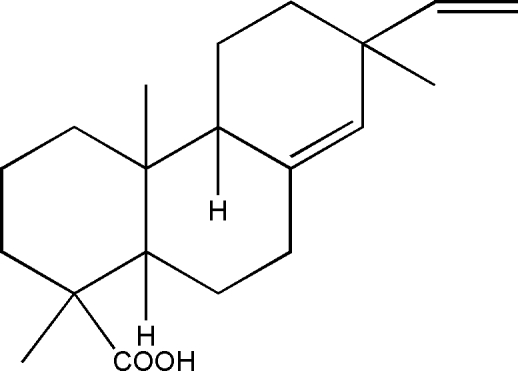


## Experimental

### 

#### Crystal data

C_20_H_30_O_2_
*M* *_r_* = 302.44
Orthorhombic, 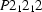

*a* = 20.818 (4) Å
*b* = 10.990 (2) Å
*c* = 7.7650 (16) Å
*V* = 1776.6 (6) Å^3^
*Z* = 4
Mo *K*α radiation
μ = 0.07 mm^−1^
*T* = 293 K
0.30 × 0.20 × 0.10 mm

#### Data collection

Enraf–Nonius CAD-4 diffractometer
Absorption correction: ψ scan (North *et al.*, 1968 ▶) *T*_min_ = 0.979, *T*_max_ = 0.993
1862 measured reflections
1862 independent reflections
1231 reflections with *I* > 2σ(*I*)
3 standard reflections every 200 reflections intensity decay: 1%

#### Refinement

*R*[*F* ^2^ > 2σ(*F* ^2^)] = 0.065
*wR*(*F* ^2^) = 0.188
*S* = 1.00
1862 reflections
193 parameters
H-atom parameters constrained
Δρ_max_ = 0.16 e Å^−3^
Δρ_min_ = −0.17 e Å^−3^

### 

Data collection: *CAD-4 EXPRESS* (Enraf–Nonius, 1994 ▶); cell refinement: *CAD-4 EXPRESS*; data reduction: *XCAD4* (Harms & Wocadlo,1995 ▶); program(s) used to solve structure: *SHELXS97* (Sheldrick, 2008 ▶); program(s) used to refine structure: *SHELXL97* (Sheldrick, 2008 ▶); molecular graphics: *SHELXTL* (Sheldrick, 2008 ▶); software used to prepare material for publication: *SHELXL97*.

## Supplementary Material

Crystal structure: contains datablocks I, global. DOI: 10.1107/S1600536809013233/at2757sup1.cif

Structure factors: contains datablocks I. DOI: 10.1107/S1600536809013233/at2757Isup2.hkl

Additional supplementary materials:  crystallographic information; 3D view; checkCIF report

## Figures and Tables

**Fig. 1.**
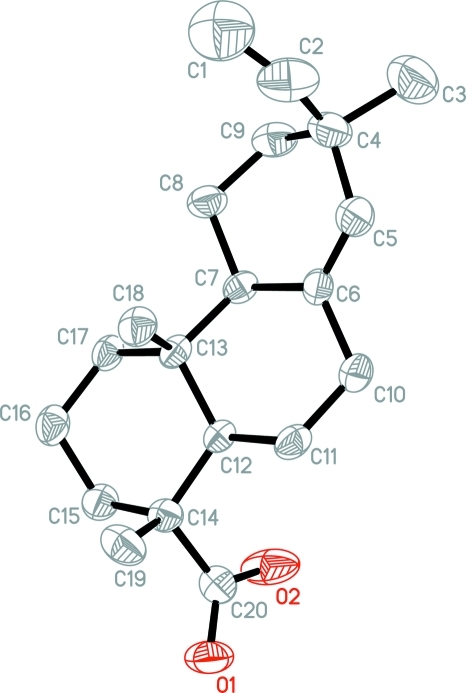
A view of the molecular structure of (I), showing displacement ellipsoids at the 30% probability level.

**Fig. 2.**
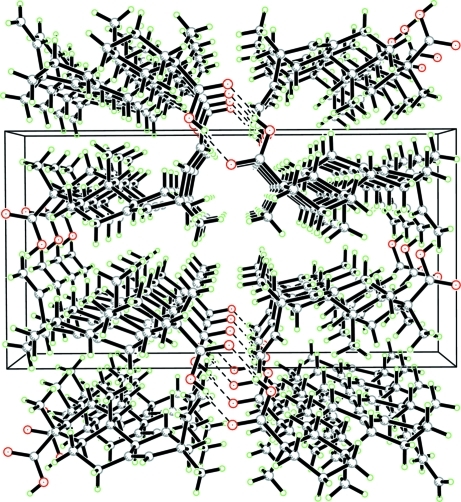
A view of the packing of the title compound.

## supplementary crystallographic information

### Comment

The title compound has been isolated from a mixture of resin acids. It was identified as pimaric acid on the basis of the comparison of its physical and spectral data with literature values (Green *et al.*, 1958; Harris *et al.*, 1948). Pimaric acid exhibits a wide range of biological activities such as trypanocidal activity(Rubio *et al.*, 2005), potent BK channel activity (Imaizumi *et al.*, 2002). Although much attention has been paid to the bioactivities of pimaric acid, the crystal structure of the title compound has not yet been reported.

In this work, we describe the crystal structure of the title compound.

The molecular structure is shown in Fig. 1 and the crystal packing in Fig.2.

The atoms of C5, C6, C7, C8 in the cyclohexene ring and the atom C10 in the conjoint cyclohexane ring are in the same plane. The two methyl groups attached to the cyclohexane rings are in axial positions and in the same direction. The crystal structure is stabilized by O2—H2B···O1 and C12—H12A···O2 hydrogen bongding interactions.

### Experimental

A mixture of resin acids and maleic anhydride were dissolved in acetic acid and the solution was refluxed for 4 h. After refluxing the solution was cooled to room temperature and then filtrated. The solvent in the filtrate was distilled away under vacuum and the remainder was dissolved in 1% sodium hydroxide solution. The solution was left standing overnight. The precipitate obtained from the solution was acidified by 5% hydrochloric acid solution and then dissolved in ether. The solution was washed with water until it was neutral, dryed with sodium sulfate and then concentrated. The residue was recrystallized with acetone and the title compound was obtained as colorless solid.

### Refinement

All H atoms bonded to the C atoms were placed geometrically at the distances of 0.93–0.98 Å and included in the refinement in riding motion approximation with *U*_iso_(H) = 1.2 or 1.5*U*_eq_ of the carrier atom.

### Crystal data

**Table:**

C_20_H_30_O_2_	*D*_x_ = 1.131 Mg m^−^^3^
*M**_r_* = 302.44	Melting point: 490K K
Orthorhombic, *P*2_1_2_1_2	Mo *K*α radiation, λ = 0.71073 Å
*a* = 20.818 (4) Å	Cell parameters from 25 reflections
*b* = 10.990 (2) Å	θ = 9–12°
*c* = 7.7650 (16) Å	µ = 0.07 mm^−^^1^
*V* = 1776.6 (6) Å^3^	*T* = 293 K
*Z* = 4	Rectangular plate, colorless
*F*(000) = 664	0.30 × 0.20 × 0.10 mm

### Data collection

**Table:**

Enraf–Nonius CAD-4 diffractometer	1231 reflections with *I* > 2σ(*I*)
Radiation source: fine-focus sealed tube	*R*_int_ = 0.0000
graphite	θ_max_ = 25.3°, θ_min_ = 2.0°
ω/2θ scans	*h* = 0→25
Absorption correction: ψ scan (North *et al.*, 1968)	*k* = 0→13
*T*_min_ = 0.979, *T*_max_ = 0.993	*l* = −9→9
1862 measured reflections	3 standard reflections every 200 reflections
1862 independent reflections	intensity decay: 1%

### Refinement

**Table:**

Refinement on *F*^2^	Primary atom site location: structure-invariant direct methods
Least-squares matrix: full	Secondary atom site location: difference Fourier map
*R*[*F*^2^ > 2σ(*F*^2^)] = 0.065	Hydrogen site location: inferred from neighbouring sites
*wR*(*F*^2^) = 0.188	H-atom parameters constrained
*S* = 1.00	*w* = 1/[σ^2^(*F*_o_^2^) + (0.1*P*)^2^ + 0.6*P*] where *P* = (*F*_o_^2^ + 2*F*_c_^2^)/3
1862 reflections	(Δ/σ)_max_ < 0.001
193 parameters	Δρ_max_ = 0.16 e Å^−^^3^
0 restraints	Δρ_min_ = −0.17 e Å^−^^3^

### Special details

**Table:**

Geometry. All e.s.d.'s (except the e.s.d. in the dihedral angle between two l.s. planes) are estimated using the full covariance matrix. The cell e.s.d.'s are taken into account individually in the estimation of e.s.d.'s in distances, angles and torsion angles; correlations between e.s.d.'s in cell parameters are only used when they are defined by crystal symmetry. An approximate (isotropic) treatment of cell e.s.d.'s is used for estimating e.s.d.'s involving l.s. planes.
Refinement. Refinement of *F*^2^ against ALL reflections. The weighted *R*-factor *wR* and goodness of fit *S* are based on *F*^2^, conventional *R*-factors *R* are based on *F*, with *F* set to zero for negative *F*^2^. The threshold expression of *F*^2^ > σ(*F*^2^) is used only for calculating *R*-factors(gt) *etc*. and is not relevant to the choice of reflections for refinement. *R*-factors based on *F*^2^ are statistically about twice as large as those based on *F*, and *R*- factors based on ALL data will be even larger.

### Fractional atomic coordinates and isotropic or equivalent isotropic displacement parameters (Å^2^)

**Table:**

	*x*	*y*	*z*	*U*_iso_*/*U*_eq_	
O1	0.99697 (17)	0.6536 (3)	0.9540 (7)	0.1009 (15)	
O2	0.91875 (17)	0.5243 (3)	0.9640 (8)	0.1170 (19)	
H2B	0.9493	0.4772	0.9603	0.176*	
C1	0.5623 (5)	0.9204 (9)	0.5670 (15)	0.149 (4)	
H1A	0.5421	0.9085	0.6725	0.179*	
H1B	0.5589	0.9950	0.5113	0.179*	
C2	0.5940 (4)	0.8361 (8)	0.5000 (12)	0.119 (3)	
H2A	0.6125	0.8558	0.3947	0.143*	
C3	0.5645 (3)	0.6180 (8)	0.4461 (11)	0.124 (3)	
H3A	0.5725	0.6340	0.3264	0.186*	
H3B	0.5200	0.6321	0.4713	0.186*	
H3C	0.5752	0.5350	0.4715	0.186*	
C4	0.6077 (3)	0.7061 (6)	0.5606 (8)	0.0801 (17)	
C5	0.6758 (2)	0.6766 (5)	0.5262 (7)	0.0711 (14)	
H5A	0.6874	0.6647	0.4118	0.085*	
C6	0.7215 (2)	0.6657 (4)	0.6420 (6)	0.0521 (11)	
C7	0.7116 (2)	0.6929 (4)	0.8315 (6)	0.0498 (11)	
H7A	0.7139	0.6144	0.8911	0.060*	
C8	0.6445 (2)	0.7431 (5)	0.8672 (7)	0.0680 (14)	
H8A	0.6339	0.7301	0.9874	0.082*	
H8B	0.6442	0.8300	0.8458	0.082*	
C9	0.5940 (2)	0.6822 (6)	0.7544 (8)	0.0787 (16)	
H9A	0.5518	0.7136	0.7837	0.094*	
H9B	0.5940	0.5952	0.7760	0.094*	
C10	0.7870 (2)	0.6163 (5)	0.5973 (6)	0.0640 (13)	
H10A	0.7920	0.6179	0.4731	0.077*	
H10B	0.7892	0.5320	0.6338	0.077*	
C11	0.8424 (2)	0.6850 (5)	0.6776 (6)	0.0577 (12)	
H11A	0.8465	0.7645	0.6245	0.069*	
H11B	0.8822	0.6409	0.6592	0.069*	
C12	0.82995 (19)	0.6994 (4)	0.8726 (5)	0.0445 (10)	
H12A	0.8203	0.6169	0.9128	0.053*	
C13	0.7675 (2)	0.7720 (4)	0.9057 (5)	0.0484 (11)	
C14	0.8910 (2)	0.7370 (4)	0.9743 (6)	0.0532 (12)	
C15	0.8742 (3)	0.7497 (5)	1.1667 (7)	0.0662 (14)	
H15A	0.8647	0.6699	1.2137	0.079*	
H15B	0.9110	0.7821	1.2280	0.079*	
C16	0.8169 (3)	0.8327 (5)	1.1954 (7)	0.0697 (14)	
H16A	0.8081	0.8385	1.3178	0.084*	
H16B	0.8270	0.9137	1.1535	0.084*	
C17	0.7580 (2)	0.7849 (5)	1.1030 (5)	0.0562 (13)	
H17A	0.7224	0.8396	1.1244	0.067*	
H17B	0.7469	0.7061	1.1505	0.067*	
C18	0.7663 (2)	0.8972 (4)	0.8218 (7)	0.0620 (13)	
H18A	0.8006	0.9458	0.8672	0.093*	
H18B	0.7260	0.9360	0.8456	0.093*	
H18C	0.7715	0.8887	0.6995	0.093*	
C19	0.9237 (2)	0.8539 (5)	0.9099 (8)	0.0758 (16)	
H19A	0.9608	0.8706	0.9794	0.114*	
H19B	0.8941	0.9206	0.9181	0.114*	
H19C	0.9366	0.8435	0.7922	0.114*	
C20	0.9393 (2)	0.6334 (4)	0.9643 (7)	0.062	

### Atomic displacement parameters (Å^2^)

**Table:**

	*U*^11^	*U*^22^	*U*^33^	*U*^12^	*U*^13^	*U*^23^
O1	0.0487 (19)	0.069 (2)	0.185 (4)	0.0073 (17)	−0.008 (3)	−0.014 (3)
O2	0.065 (2)	0.063 (2)	0.223 (6)	0.0112 (18)	−0.001 (3)	0.030 (3)
C1	0.150 (8)	0.132 (7)	0.166 (10)	0.000 (6)	−0.001 (8)	0.027 (7)
C2	0.098 (5)	0.129 (7)	0.130 (7)	0.024 (5)	−0.018 (5)	0.027 (6)
C3	0.090 (5)	0.158 (7)	0.124 (6)	−0.019 (5)	−0.015 (5)	−0.021 (6)
C4	0.056 (3)	0.104 (5)	0.080 (4)	0.003 (3)	−0.012 (3)	−0.004 (4)
C5	0.065 (3)	0.086 (4)	0.062 (3)	0.001 (3)	0.007 (3)	−0.009 (3)
C6	0.056 (3)	0.049 (2)	0.051 (3)	0.000 (2)	0.004 (2)	−0.009 (2)
C7	0.050 (3)	0.052 (3)	0.048 (2)	0.007 (2)	0.010 (2)	0.007 (2)
C8	0.049 (3)	0.088 (3)	0.067 (3)	0.012 (3)	0.004 (3)	−0.003 (3)
C9	0.053 (3)	0.084 (4)	0.100 (4)	0.003 (3)	0.004 (3)	0.008 (4)
C10	0.064 (3)	0.076 (3)	0.052 (3)	0.008 (3)	0.008 (2)	−0.013 (3)
C11	0.051 (3)	0.069 (3)	0.053 (3)	0.010 (2)	0.014 (2)	−0.002 (3)
C12	0.049 (2)	0.042 (2)	0.042 (2)	0.0070 (19)	0.007 (2)	0.005 (2)
C13	0.055 (2)	0.050 (2)	0.041 (2)	0.013 (2)	0.004 (2)	0.001 (2)
C14	0.052 (2)	0.045 (2)	0.063 (3)	0.002 (2)	−0.004 (2)	0.006 (2)
C15	0.071 (3)	0.070 (3)	0.057 (3)	0.016 (3)	−0.011 (3)	0.000 (3)
C16	0.089 (4)	0.069 (3)	0.052 (3)	0.017 (3)	−0.009 (3)	−0.008 (3)
C17	0.068 (3)	0.059 (3)	0.042 (3)	0.016 (2)	0.001 (2)	−0.004 (2)
C18	0.073 (3)	0.051 (3)	0.062 (3)	0.010 (2)	−0.006 (3)	0.007 (2)
C19	0.070 (3)	0.065 (3)	0.093 (4)	−0.013 (3)	−0.021 (3)	0.011 (3)
C20	0.068	0.053	0.064	−0.002	−0.007	0.008

### Geometric parameters (Å, °)

**Table:**

O1—C20	1.223 (6)	C10—H10A	0.9700
O2—C20	1.273 (6)	C10—H10B	0.9700
O2—H2B	0.8200	C11—C12	1.544 (6)
C1—C2	1.251 (11)	C11—H11A	0.9700
C1—H1A	0.9300	C11—H11B	0.9700
C1—H1B	0.9300	C12—C13	1.546 (6)
C2—C4	1.531 (10)	C12—C14	1.552 (6)
C2—H2A	0.9300	C12—H12A	0.9800
C3—C4	1.592 (9)	C13—C18	1.523 (6)
C3—H3A	0.9600	C13—C17	1.551 (6)
C3—H3B	0.9600	C14—C20	1.520 (6)
C3—H3C	0.9600	C14—C19	1.538 (6)
C4—C5	1.479 (7)	C14—C15	1.541 (7)
C4—C9	1.554 (8)	C15—C16	1.517 (7)
C5—C6	1.315 (6)	C15—H15A	0.9700
C5—H5A	0.9300	C15—H15B	0.9700
C6—C10	1.507 (6)	C16—C17	1.515 (7)
C6—C7	1.516 (6)	C16—H16A	0.9700
C7—C8	1.527 (6)	C16—H16B	0.9700
C7—C13	1.563 (6)	C17—H17A	0.9700
C7—H7A	0.9800	C17—H17B	0.9700
C8—C9	1.524 (7)	C18—H18A	0.9600
C8—H8A	0.9700	C18—H18B	0.9600
C8—H8B	0.9700	C18—H18C	0.9600
C9—H9A	0.9700	C19—H19A	0.9600
C9—H9B	0.9700	C19—H19B	0.9600
C10—C11	1.514 (7)	C19—H19C	0.9600
			
C20—O2—H2B	109.5	C12—C11—H11B	109.9
C2—C1—H1A	120.0	H11A—C11—H11B	108.3
C2—C1—H1B	120.0	C11—C12—C13	110.9 (3)
H1A—C1—H1B	120.0	C11—C12—C14	112.8 (4)
C1—C2—C4	131.5 (10)	C13—C12—C14	117.8 (3)
C1—C2—H2A	114.3	C11—C12—H12A	104.6
C4—C2—H2A	114.3	C13—C12—H12A	104.6
C4—C3—H3A	109.5	C14—C12—H12A	104.6
C4—C3—H3B	109.5	C18—C13—C12	114.1 (4)
H3A—C3—H3B	109.5	C18—C13—C17	109.7 (4)
C4—C3—H3C	109.5	C12—C13—C17	108.6 (4)
H3A—C3—H3C	109.5	C18—C13—C7	109.4 (4)
H3B—C3—H3C	109.5	C12—C13—C7	106.2 (3)
C5—C4—C2	109.1 (6)	C17—C13—C7	108.7 (4)
C5—C4—C9	108.3 (5)	C20—C14—C19	108.4 (4)
C2—C4—C9	114.9 (6)	C20—C14—C15	105.6 (4)
C5—C4—C3	107.9 (5)	C19—C14—C15	109.9 (4)
C2—C4—C3	106.9 (6)	C20—C14—C12	108.5 (4)
C9—C4—C3	109.5 (6)	C19—C14—C12	114.8 (4)
C6—C5—C4	126.2 (5)	C15—C14—C12	109.3 (4)
C6—C5—H5A	116.9	C16—C15—C14	112.1 (4)
C4—C5—H5A	116.9	C16—C15—H15A	109.2
C5—C6—C10	122.0 (4)	C14—C15—H15A	109.2
C5—C6—C7	123.1 (4)	C16—C15—H15B	109.2
C10—C6—C7	114.7 (4)	C14—C15—H15B	109.2
C6—C7—C8	111.8 (4)	H15A—C15—H15B	107.9
C6—C7—C13	111.4 (4)	C17—C16—C15	111.0 (4)
C8—C7—C13	114.4 (4)	C17—C16—H16A	109.4
C6—C7—H7A	106.2	C15—C16—H16A	109.4
C8—C7—H7A	106.2	C17—C16—H16B	109.4
C13—C7—H7A	106.2	C15—C16—H16B	109.4
C9—C8—C7	111.6 (4)	H16A—C16—H16B	108.0
C9—C8—H8A	109.3	C16—C17—C13	113.4 (4)
C7—C8—H8A	109.3	C16—C17—H17A	108.9
C9—C8—H8B	109.3	C13—C17—H17A	108.9
C7—C8—H8B	109.3	C16—C17—H17B	108.9
H8A—C8—H8B	108.0	C13—C17—H17B	108.9
C8—C9—C4	110.8 (5)	H17A—C17—H17B	107.7
C8—C9—H9A	109.5	C13—C18—H18A	109.5
C4—C9—H9A	109.5	C13—C18—H18B	109.5
C8—C9—H9B	109.5	H18A—C18—H18B	109.5
C4—C9—H9B	109.5	C13—C18—H18C	109.5
H9A—C9—H9B	108.1	H18A—C18—H18C	109.5
C6—C10—C11	114.5 (4)	H18B—C18—H18C	109.5
C6—C10—H10A	108.6	C14—C19—H19A	109.5
C11—C10—H10A	108.6	C14—C19—H19B	109.5
C6—C10—H10B	108.6	H19A—C19—H19B	109.5
C11—C10—H10B	108.6	C14—C19—H19C	109.5
H10A—C10—H10B	107.6	H19A—C19—H19C	109.5
C10—C11—C12	109.1 (4)	H19B—C19—H19C	109.5
C10—C11—H11A	109.9	O1—C20—O2	120.0 (5)
C12—C11—H11A	109.9	O1—C20—C14	121.1 (4)
C10—C11—H11B	109.9	O2—C20—C14	118.8 (4)
			
C1—C2—C4—C5	−138.0 (10)	C14—C12—C13—C7	−163.7 (4)
C1—C2—C4—C9	−16.1 (13)	C6—C7—C13—C18	66.0 (5)
C1—C2—C4—C3	105.6 (12)	C8—C7—C13—C18	−62.1 (5)
C2—C4—C5—C6	108.1 (7)	C6—C7—C13—C12	−57.6 (4)
C9—C4—C5—C6	−17.6 (9)	C8—C7—C13—C12	174.3 (4)
C3—C4—C5—C6	−136.1 (6)	C6—C7—C13—C17	−174.2 (4)
C4—C5—C6—C10	170.2 (5)	C8—C7—C13—C17	57.7 (5)
C4—C5—C6—C7	−5.4 (9)	C11—C12—C14—C20	−65.6 (5)
C5—C6—C7—C8	−4.4 (7)	C13—C12—C14—C20	163.0 (4)
C10—C6—C7—C8	179.7 (4)	C11—C12—C14—C19	55.8 (5)
C5—C6—C7—C13	−133.9 (5)	C13—C12—C14—C19	−75.5 (5)
C10—C6—C7—C13	50.2 (5)	C11—C12—C14—C15	179.8 (4)
C6—C7—C8—C9	37.2 (6)	C13—C12—C14—C15	48.4 (5)
C13—C7—C8—C9	165.1 (4)	C20—C14—C15—C16	−168.9 (4)
C7—C8—C9—C4	−61.6 (6)	C19—C14—C15—C16	74.3 (5)
C5—C4—C9—C8	49.7 (7)	C12—C14—C15—C16	−52.4 (6)
C2—C4—C9—C8	−72.5 (6)	C14—C15—C16—C17	59.3 (6)
C3—C4—C9—C8	167.2 (5)	C15—C16—C17—C13	−58.9 (6)
C5—C6—C10—C11	137.5 (5)	C18—C13—C17—C16	−74.5 (5)
C7—C6—C10—C11	−46.6 (6)	C12—C13—C17—C16	50.8 (5)
C6—C10—C11—C12	50.2 (6)	C7—C13—C17—C16	165.9 (4)
C10—C11—C12—C13	−60.7 (5)	C19—C14—C20—O1	18.5 (7)
C10—C11—C12—C14	164.6 (4)	C15—C14—C20—O1	−99.2 (6)
C11—C12—C13—C18	−56.5 (5)	C12—C14—C20—O1	143.8 (5)
C14—C12—C13—C18	75.7 (5)	C19—C14—C20—O2	−160.4 (5)
C11—C12—C13—C17	−179.2 (4)	C15—C14—C20—O2	81.9 (6)
C14—C12—C13—C17	−47.0 (5)	C12—C14—C20—O2	−35.2 (7)
C11—C12—C13—C7	64.1 (4)		

## References

[bb1] Enraf–Nonius (1994). *CAD-4 Software* Enraf–Nonius, Delft, The Netherlands.

[bb2] Green, B., Harris, A. & Whalley, W. B. (1958). *J. Chem. Soc.* pp. 4715–4719.

[bb3] Harms, K. & Wocadlo, S. (1995). *XCAD4* University of Marburg, Germany.

[bb4] Harris, G. C. & Sanderson, T. F. (1948). *J. Am. Chem. Soc.* **70**, 2081–2085. 10.1021/ja01186a029 18863802

[bb5] Imaizumi, Y., Sakamoto, K., Yamada, A., Hotta, A., Ohya, S., Muraki, K., Uchiyama, M. & Ohwada, T. (2002). *Mol. Pharmacol.* **62**, 836–846. 10.1124/mol.62.4.836 12237330

[bb6] North, A. C. T., Phillips, D. C. & Mathews, F. S. (1968). *Acta Cryst.* A**24**, 351–359.

[bb7] Rubio, J., Calderon, J. S., Flores, A., Castroa, C. & Cespedes, C. L. (2005). *J. Biosci.* **60**, 711–716. 10.1515/znc-2005-9-1009 16320613

[bb8] Sheldrick, G. M. (2008). *Acta Cryst.* A**64**, 112–122. 10.1107/S0108767307043930 18156677

